# ‘I don’t think I’d be frightened if the statins went’: a phenomenological qualitative study exploring medicines use in palliative care patients, carers and healthcare professionals.

**DOI:** 10.1186/s12904-016-0086-7

**Published:** 2016-01-29

**Authors:** Adam Todd, Holly Holmes, Sallie Pearson, Carmel Hughes, Inga Andrew, Lisa Baker, Andy Husband

**Affiliations:** Division of Pharmacy, School of Medicine, Pharmacy and Health, Durham University, Queen’s Campus, University Boulevard, Thornaby, Stockton-on-Tees TS17 6BH UK; Division of Geriatric and Palliative Medicine, The University of Texas Health Science Center, Houston, TX USA; Faculty of Pharmacy and School of Public Health, The University of Sydney, Sydney, Australia; School of Pharmacy, Queen’s University, Belfast, UK; St Benedict’s Hospice and Centre for Specialist Palliative Care, Ryhope, Sunderland, UK

**Keywords:** Deprescribing, Phenomenology, Polypharmacy, Palliative care

## Abstract

**Background:**

There is a growing body of evidence suggesting patients with life-limiting illness use medicines inappropriately and unnecessarily. In this context, the perspective of patients, their carers and the healthcare professionals responsible for prescribing and monitoring their medication is important for developing deprescribing strategies. The aim of this study was to explore the lived experience of patients, carers and healthcare professionals in the context of medication use in life-limiting illness.

**Methods:**

In-depth interviews, using a phenomenological approach: methods of transcendental phenomenology were used for the patient and carer interviews, while hermeneutic phenomenology was used for the healthcare professional interviews.

**Results:**

The study highlighted that medication formed a significant part of a patient’s day-to-day routine; this was also apparent for their carers who took on an active role-as a gatekeeper of care-in managing medication. Patients described the experience of a point in which, in their disease journey, they placed less importance on taking certain medications; healthcare professionals also recognize this and refer it as a ‘transition’. This point appeared to occur when the patient became accepting of their illness and associated life expectancy. There was also willingness by patients, carers and healthcare professionals to review and alter the medication used by patients in the context of life-limiting illness.

**Conclusions:**

There is a need to develop deprescribing strategies for patients with life-limiting illness. Such strategies should seek to establish patient expectations, consider the timing of the discussion about ceasing treatment and encourage the involvement of other stakeholders in the decision-making progress.

## Background

Improving care for people with life-limiting illness is an international priority [1]. Due to aging populations, and the increasing proportion of the population with advanced illness at the end of life, palliative care is a major public health issue and forms a key part of our society [2]. One important component of this agenda-given that many patients with advanced illness have other complex, co-morbid conditions-is ensuring their pharmacotherapy is appropriate, safe and rational. There is, however, growing evidence suggesting patients with life-limiting illness use an excess number of medications, many of which are inappropriate and unnecessary-this contributes to a high pill burden for the patient and, in some cases, has significant safety implications [3–7]. In view of this, there is a movement towards developing ‘deprescribing’ approaches for these patients, that is the process of reducing or discontinuing drugs, aimed at minimizing polypharmacy and improving patient outcomes. For example, Scott and colleagues have developed a five step deprescribing protocol that considers, amongst other things, indication of the medication; the benefit-harm ratio and the potential harm or burden of future treatment [8]. Lindsay and colleagues have taken a different approach and produced a deprescribing guideline suitable for use in palliative cancer patients; [9] this is a defined list of medications (or classes of medication) that could be suitable for stopping in this population (e.g. the use of aspirin for primary prevention of cardiovascular disease). However, despite these approaches, patients continue to be prescribed medication unnecessarily and inappropriately-the reasons for which are unclear. To take a holistic approach to developing deprescribing strategies, it is important to understand the perspective of patients, their carers and the healthcare professionals responsible for prescribing their medication-something which current strategies fail to do. Unfortunately, there is a scarcity of literature in this area and little is known about how these groups experience pharmacological care in the context of life-limiting illness. This study, therefore, aimed to explore the lived experience of patients, carers and healthcare professionals in the context of medication use in life-limiting illness.

## Method

### Recruitment and sampling

Patients attending a day care centre at a specialist palliative care unit based within the North of England were invited to take part in the study. The day care centre is a small, modern purpose built facility; it accommodates around ten patients in any one time and offers clinical outpatient services, as well as providing social activities for patients. The average life expectancy of patients attending the day care centre was approximately 18 months. To be included in the study, patients and carers had to be over 18 years of age and healthcare professionals had to be responsible for prescribing medication to this general patient group. Carers were identified and approached through patients attending the day care centre. For the purposes of the study, a carer was defined as a friend or family member supporting the needs of a patient with life-limiting illness. Carers employed by the National Health Service (NHS) or charities in that capacity were excluded from the study. A purposive sampling framework was used to ensure there was maximum variation in gender, age and type of life limiting illness for the patient and carer participants and by type of practitioner for the healthcare professional participants. Recruitment ended once theoretical data saturation was reached, i.e. when no new themes emerged, across the three constituent groups, as assessed by two researchers (AT and AH).

### Methodological approach

In-depth interviews, using a phenomenological approach, were used to explore the ‘lived experiences’ of patients and their carers, as well as those healthcare professionals who are involved in prescribing and monitoring medication for patients with life-limiting illness. Methods of transcendental phenomenology [10] were used for the patient and carer interviews to explore experiences of medication use; i.e. ‘what’ was experienced, as well as the way in which care was provided i.e. ‘how’ medication use was experienced. Transcendental phenomenology is focused on describing experiences through the eyes of participants, rather than presenting interpretative representations. Accordingly, any prior beliefs or knowledge about medication use for this group were ‘bracketed’ by the interviewer, enabling the accounts of patients and carers to be told without influence, thus appearing fresh. Given the complexities of prescribing medication to patients with life-limiting illness, and the need for healthcare professionals to engage in evidence-based care, methods of hermeneutic (or existential) phenomenology [11] were used for these interviews. Hermeneutic phenomenology focuses upon the interpretation of an experience; the methodology recognizes the context within which experiences take place and uses this to better understand the lived experience. Hermeneutic phenomenology is appropriate in this context, as it allows for subtle interpretations and allows for discussions about evidence-based practice set against the realities of day-to-day care. Given the hermeneutic phenomenological approach to these interviews, they were informed by the previous interviews with patients and carers.

Interviews with patients were conducted in a specialist palliative care unit within an outpatient setting; interviews with carers were conducted in their own home, while interviews with healthcare professionals were conducted in their place of work. The interviews for patients and carers were undertaken separately.

During the interview, participants were asked three open-ended questions:

- What is a normal day like for you?
- What are your experiences of medication use (from the perspective of a patient/carer/healthcare professional)?
- Is there any desire for change?

**Fig. 1:**
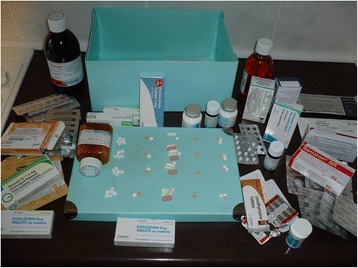
The image shown to participants during the interview

Each question was followed up with probing questions to explore the participants’ lived experience. Towards the end of each interview, an image of multiple medications was shown to participants to help understand their experiences (Fig. 1). This technique, known as photo elicitation, is a recognized method in qualitative research [12] and can elicit more information from the participant, as parts of the brain that process visual information are older in evolutionary terms than parts that process verbal information [13].

### Data analysis

Interviews were recorded, transcribed and analyzed verbatim by two researchers (AT and AH) using thematic analysis with the following stages: familiarization with the data by re-reading transcripts; identification of significant phrases that pertained to the experience; formulating meaning of the phrases and clustering those themes common to all of the transcripts; integrating the themes into an in-depth, exhaustive description of the phenomenon [14]. Any discrepancies were resolved through discussion (AT, AH) and if agreement was not reached, by consensus (IA). Emergent themes were tested using diverse accounts between cases, in order to challenge the integrity of the boundaries of themes. Data analysis produced a textural description of ‘what’ the participants experienced and expected, as well as a structural description of ‘how’ they experienced pharmacological care: the combination of the two conveyed their overall experience. When using direct quotes from patients and carers, pseudonyms were given to ensure confidentiality.

### Ethical approval

The study complied with the 2013 Declaration of Helsinki; ethical approval was obtained from Durham University (reference ESC2/2013/17). All participants provided written informed consent prior to participating in the study.

## Results

Thirty-six participants in total were recruited to the study: 12 for each group. The characteristics for each patient are described in Table 1; for the healthcare professional interviews, three palliative medicine consultants, three advanced nurse practitioners and six general practitioners (GPs) were recruited. All of the carers recruited to the study were family members of the patient. From the interviews, themes specifically emerged around medication use, which fell into three main categories: medication as part of daily routine; risks of medication; and, willingness to change.

**Table 1:** Patient participant characteristics

Participant number	Gender	Age range in years	Life limiting illness
1	Male	51–60	Lung Cancer
2	Female	61–70	Liver Cancer
3	Male	61–70	Parkinson’s Disease
4	Male	71–79	Prostate Cancer
5	Male	61–70	Prostate Cancer
6	Female	71–79	COPD
7	Male	51–60	Prostate Cancer
8	Male	<50	Motor Neurone Disease
9	Female	51–60	Colorectal Cancer
10	Female	71–79	Renal Cancer
11	Female	≥80	Heart failure
12	Male	≥80	COPD

### Medication forms part of daily routine

The majority of patient and carer participants specifically referred to medication when asked to describe what a normal day was like for them. Many patient participants described the daily routine of organizing and taking the medication, while carers often referred to organizing patients’ medication, including following-up medication-related changes with the GP. One patient used the term ‘habit’ to describe their experience of taking medication. Carers described acting as gatekeepers in this context: they felt compelled to take ownership and responsibility for managing patients’ medication.

*Well, on a Saturday morning it’s the drug day. And I’m in the kitchen for half-an-hour with all the boxes and, you know. I go through the medication, put them in the boxes and I’m checking to see if we need any, and if we need any I have the reserve supply elsewhere in the dining room, and I’ll go and get them from there.* Dorothy, carer.

When describing their medication regimen, several patients revealed they did not know what particular medications they used, or the indications for the medication. This knowledge was not important to patients, as they had complete trust in the healthcare professionals responsible for their care.

*Well doctor says to me you take that tablet, that tablet, that tablet, end of story. What the man says, you take. There’s no good saying why, that one’s for your liver, that one’s for your heart and that one’s for your whatever, and stuff like that. Just take them and Bob’s your uncle.* Donald, Parkinson’s disease patient

This lack of understanding was not experienced by carers, who had a good knowledge and understanding of the patient’s medication; this appeared to stem from the feeling of having responsibility for the patients’ medication.

When responding to the photograph of multiple medications, healthcare professionals stated that the image was very familiar and, although they were dismayed by it, it was acknowledged that the challenges associated with taking multiple medications were part of their daily routine when managing patients with life limiting illness.

*Familiarity! Absolutely total and utter confusion and a huge risk for error all along the way for the patient-how on earth the patient gets his head round taking, or her head round taking all of that lot, I really have no idea, and that’s something I’ve been very aware of when I’ve been out to homes.* General Practitioner 6

### Risk of medication

The majority of patients described experiencing adverse effects from taking their medications; this appeared to form a significant part of the overall experience of using medications. It was also acknowledged by participants that no medication was fully ‘safe’ and entirely free from risk. The adverse effects described by patients were from a mixture of medication types, including those that were disease-modifying (e.g. chemotherapy), or medications used to treat or prevent long-term condition (e.g. anti-hypertensives). Patients also felt as though the perceived risk and benefits of taking specific medication changed, depending on where they were within the journey of their disease: a state of anxiety was described until a specific point in the journey was reached. This point was described after the patient was diagnosed with life-limiting illness and appeared to occur when the patient was accepting of their disease.

*Well, I used to have high blood pressure, hypertension. I used to check my blood pressure every day and religiously take my tablets. And I thought eh, and I would say it is higher than yesterday, and this used to worry me. Now I don’t worry about it; I don’t even check-I still take my blood pressure tablets-but not religiously like I used to. It’s not so important.* Martha, liver cancer patient

Healthcare professionals also described experiencing this change where patients placed less importance on certain medications. Such a phenomenon was referred to as a ‘transition’ in which it was acknowledged that patients placed less importance on certain medications. This term was analogous to the experience described by patients, where anxiety was described until a specific point in the treatment journey was reached. In some cases, healthcare professionals appeared to respond to this state and modified their treatment approach to account for this.

*Apart from a tiny percentage of people who perhaps have an unrealistic expectation about what the medication can do, and my experience of patients dealing with, you know, patients in palliative care is that most transition beautifully, most can do it, most don’t worry about it.* Palliative Medicine Consultant (3)

Patients perceived that not all medication was the same and placed different values and beliefs on them in terms of their risk and benefit. These values and beliefs were not consistent and varied between types of medication. The experience of taking statins was often described:

*I was put on a statin and I didn’t like it. It was giving me physical side effects. I wanted to stop my statin but I thought I’d be committing suicide – I know it’s important for my heart.* William, prostate cancer

*I don’t think I’d be frightened if the statins went. And I think the medical profession as well often make mistakes about good cholesterol and bad cholesterol. You know, some cholesterol is actually good for you and I think too many doctors, just say on a high cholesterol reading, terrible, get out the statins.* George, prostate cancer

### Willingness to change medication

Across all participant groups, there was an acknowledgement that medications were burdensome interventions and there was a willingness to rationalize them in this context. This was particularly the case where treatment regimens had a high pill burden. In many cases, patients were not concerned with the type of the medication they were taking, but were overwhelmed by the volume of it-one patient described this experience as ‘disgusting’. This was further exacerbated when patients had difficulty in swallowing medication.

*I cannot swallow them, you know if they’re coated, like a Smartie, I’m okay, but if they’re not coated I cannot them, cannot get them down. So when it like sort of melts in your mouth that’s when I feel sick. And I’ll say oh I’m not taking them. I’m always changing, telling the doctors like.* Annie, heart failure patient

In this context, the acknowledgement by participants that medications were burdensome, both in terms of volume and type appeared to act as an impetus for wanting change. Carers would embrace deprescribing approaches, providing the risks and benefits were properly explained and it was done for the benefit of the patient.

*If stopping the tablets are for her benefit then I’m happy with that. But if I thought it was for costs or anything like that, or trying to make the books budget I wouldn’t be happy with that at all.* Jenny, carer

Given the complexity of care for patients with life-limiting illness, healthcare professionals found communicating with each other frustrating and this was acknowledged as a barrier to change; this challenge was particularly evident for the interfaces between primary, secondary and tertiary care. The importance of coming to a joint decision between healthcare professional, patient and carer was perceived as important by all participants when considering deprescribing medication.

*I’ve had GPs that I’ve stopped statins and then they’ve been restarted and I haven’t known if it’s because it’s on the repeat prescription and they haven’t stopped it, and then that’s me back on the phone-can you stop the statin.* Palliative Care Consultant 1

Despite these challenges, all of the participants did, however, have experience of-in terms of giving, observing or receiving-a deprescribing event. In some cases, when medication was initiated, patients were told that they would be taking it ‘for the rest of their life’; this was literally interpreted by patients that they would be taking the medication until the day they died. The experience of being told this appeared to be a significant barrier to deprescribing approaches, as it created a mismatch of expectations between healthcare professional and patient and carer regarding treatment.

*I did used to take blood pressure tablets atenolol and lisinopril, and was told by the GP I used to see oh you’ll be on these for the rest of your life. And my current GP, she’s looked at it said, oh I am going to take you off the blood pressure tablets. I wasn’t scared but I was surprised.* George, prostate cancer patient

In some cases, when instigating a deprescribing event, the healthcare professional was required to have discussions with patient and carer, regarding treatment expectations and disease trajectory. Several healthcare professionals described discussions of this nature as ‘difficult’ and considered that appropriate timing of these discussions was crucial.

## Discussion

This is the first study to describe the lived experience of patients, carers and healthcare professionals in the context of medication use in life limiting illness. The study highlighted that medication formed a significant part of a patient’s day-to-day routine; this was also apparent for their carers who took on an active role-as a gatekeeper of care-in managing medication. Patients also described a phenomenon of experiencing anxiety until a specific point in the treatment journey was reached, while healthcare professionals also recognized this and described it as a ‘transition’ where the patient places less importance on taking certain medications. This phenomenon was described after the patient was diagnosed with a life-limiting illness and appeared to occur once the patient became accepting of their illness and associated life expectancy. The medications associated with this ‘transition’ were typically used to treat or prevent long-term conditions and had been taken chronically by patients for a number of years. There was also willingness by all participants to change the amount of medication that was used in the context of life-limiting illness; this change was related to the type of medication but, more importantly, for many patients, the pill burden.

There have been several other studies exploring the perceptions of patients with diminished life expectancy regarding medication [15–18]. Of note, Sand and colleagues [16] explored medication use in a group of patients with advanced cancer, and showed there was a desire to reduce the number of tablets they took, as the medication reminded them of their illness, while Qi and colleagues [17] showed the majority of older people were accepting of having one of their medications deprescribed. Tjia and colleague [19] assessed the applicability of a conceptual carer medication management skills framework [20], by analyzing home hospice visits between a nurse, patient and carer. The themes that emerged in this work-such as medication knowledge and discontinuing medications-were similar to what is reported in our sample of participants, which lends support to our findings.

In terms of implications for policy, many patients in our study described positive experiences of deprescribing-particularly with regards to medication used to treat or prevent long-term conditions. The finding is timely given a recent randomized trial on statin discontinuation which has shown that stopping statins in patients with anticipated life expectancy from one month to one year did not adversely affect patient outcomes and indeed, patients who stopped statins had a better quality of life [21]. Our work shows that when approaching a deprescribing intervention for a patient with life-limiting illness, a number of critical points should be considered. Firstly, establishing the patient expectations of their medication, as some patients may believe that they will be taking specific medication until the day they die. This has recently been highlighted in a set of recommendations to support deprescribing approaches in life-limiting illness [22]. The second factor is the timing of the discussion regarding a deprescribing event, which is important, as our work shows there is a transition in which a patient places less importance on taking certain medication. Discussing a deprescribing intervention with a patient who has not yet reached this point is likely to result in a poor outcome. The third factor that appears to be important is encouraging the involvement of other stakeholders in the decision-making process, as carers (such as family members) also have an active involvement in the management of medication. These critical points should be considered in any wider approach to developing future deprescribing interventions in this population. It is interesting to note that current deprescribing approaches directed towards this population do not appear to consider these critical points.

Good communication between healthcare professionals enabling good information transfer was also considered important when deprescribing medication-something which has been acknowledged [23]. Previous qualitative work has shown that GPs were anxious about discontinuing preventative medication in very old patients, as they worried patients may have interpreted this as a sign of being ‘abandoned’ [24]. Importantly, our work does not support this, but instead shows that deprescribing medication-or broaching the subject of deprescribing medication-does not appear to affect the hope of patient in terms of their illness.

While we believe our results are robust and have important implications for the way in which medication is prescribed to patients with life-limiting illness, we do acknowledge that the research was predominantly focused on a small sample of people in the North of England. We did not assess or sample patient or carer participants by socioeconomic status or ethnicity. It is possible that participants with different ethnicities or different socioeconomic classes may have different experiences of using medication in the context of life limiting illness. Our results should therefore be interpreted with this in mind. Given the role as carers as gatekeepers of healthcare, and that few studies have explored the experience of carers alongside patients and healthcare professionals, we acknowledge this as a key strength of our study. As part of our methodology, we interviewed patients and carers separately to ensure a true description of their lived experience was obtained; interviewing patients and carers together may elicit different responses and this could be an avenue for future research.

Our methodological approach also had strengths and weaknesses: as part of our interview, we showed participants-as a method of photo elicitation-an image of medications. This approach strengthens this study, as images tend to evoke deeper elements of the human conscious than words and accordingly can yield more descriptive data. In terms of weaknesses, while phenomenology has been successfully employed as a methodology to improve understanding of the experience of illness [25–27], we do acknowledge that this method requires participants to describe the nature of their experience. Several patient participants struggled to do this and instead provided a narrative of their illness, rather than focus on the experience of it.

## Conclusion

There is willingness-from the lived experience of patients, carers and healthcare professionals-to change the amount of medication used by patients in the context of life-limiting illness. There is, therefore, a need to develop deprescribing strategies for patients with life-limiting illness. Such strategies should seek to establish patient expectations of medication, consider the timing of the discussion, and encourage the involvement of other stakeholders in the decision-making progress.

## References

[1] Hall S, Petkova H, Tsouros AD, Costantini M, Higginson IJ. Palliative care for older people: better practices. World Health Organisation. 2011. Available at: http://www.euro.who.int/__data/assets/pdf_file/0017/143153/e95052.pdf. Accessed 14 Oct 2015.

[2] Stjernswärd J, Foley KM, Ferris FD (2007). The public health strategy for palliative care. J Pain Symptom Manage.

[3] Todd A, Nazar H, Pearson H, Andrew L, Baker L, Husband A (2014). Inappropriate prescribing in patients accessing specialist palliative day care services. Int J Clin Pharm.

[4] Todd A, Williamson S, Husband A, Baqir W, Mahony M (2013). Patients with advanced lung cancer: is there scope to discontinue inappropriate medication?. Int J Clin Pharm.

[5] Tjia J, Briesacher BA, Peterson D, Liu Q, Andrade SE, Mitchell SL (2014). Use of medications of questionable benefit in advanced dementia. JAMA Intern Med.

[6] Todd A, Husband A, Andrew I, Pearson S, Lindsey L, Holmes H. Inappropriate prescribing of preventative medication in patients with life-limiting illness: a systematic review. BMJ Support Palliat Care. 2016. doi:10.1136/bmjspcare-2015-000941. 10.1136/bmjspcare-2015-000941 26733578

[7] Kotlinska-Lemieszek A, Paulsen O, Kaasa S (2014). Polypharmacy in patients with advanced cancer and pain: a european cross-sectional study of 2282 patients. J Pain Symptom Manage.

[8] Scott IA, Hilmer SN, Reeve E, Potter K, Le Couteur D, Rigby D (2015). Reducing inappropriate polypharmacy: the process of deprescribing. JAMA Intern Med.

[9] Lindsay J, Dooley M, Martin J, Fay M, Kearney A, Khatun M (2015). The development and evaluation of an oncological palliative care deprescribing guideline: the 'OncPal deprescribing guideline'. Support Care Cancer.

[10] Husserl E (1970). The crisis of European sciences and transcendental phenomenology.

[11] Van Manen M (1990). Researching the lived experience: human science for an action sensitive pedagogy.

[12] Close H (2007). The use of photography as a qualitative research tool. Nurse Res.

[13] Harper D (2002). Talking about pictures: a case for photo elicitation. Vis Stud.

[14] Cresswell JW (2006). Qualitative inquiry and research design: choosing among five approaches.

[15] Murray SA, Boyd K, Kendall M, Worth A, Benton TF, Clausen H (2002). Dying of lung cancer or cardiac failure: prospective qualitative interview study of patients and their carers in the community. BMJ.

[16] Sand AM, Harris J, Rosland JH (2009). Living with advanced cancer and short life expectancy: patients' experiences with managing medication. J Palliat Care.

[17] Qi K, Reeve E, Hilmer SN, Pearson SA, Matthews S, Gnjidic D (2015). Older peoples' attitudes regarding polypharmacy, statin use and willingness to have statins deprescribed in Australia. Int J Clin Pharm.

[18] McKechnie R, MacLeod R, Keeling S (2007). Facing uncertainty: the lived experience of palliative care. Palliat Support Care.

[19] Tjia J, Ellington L, Clayton MF, Lemay C, Reblin M (2015). Managing medications during home hospice cancer care: the needs of family caregivers. J Pain Symptom Manage.

[20] Lau DT, Kasper JD, Hauser JM, Berdes C, Chang CH, Berman RL (2009). Family caregiver skills in medication management for hospice patients: a qualitative study to define a construct. J Gerontol B Psychol Sci Soc Sci.

[21] Kutner JS, Blatchford PJ, Taylor DH, Ritchie CS, Bull JH, Fairclough DL (2015). Safety and benefit of discontinuing statin therapy in the setting of advanced, life-limiting illness: a randomized clinical trial. JAMA Intern Med.

[22] Todd A, Holmes HM. Recommendations to support deprescribing medications late in life. Int J Clin Pharm. 10.1007/s11096-015-0148-6. 10.1007/s11096-015-0148-6 PMC4682203 26078120

[23] Anderson K, Stowasser D, Freeman C, Scott I (2014). Prescriber barriers and enablers to minimising potentially inappropriate medications in adults: a systematic review and thematic synthesis. BMJ Open.

[24] Schuling J, Gebben H, Veehof LJ, Haaijer-Ruskamp FM (2012). Deprescribing medication in very elderly patients with multimorbidity: the view of Dutch GPs. A qualitative study.. BMC Fam Pract.

[25] Haddow G (2005). The phenomenology of death, embodiment and organ transplantation. Sociol Health Illn.

[26] Miller C, Jezewski MA (2001). A phenomenologic assessment of relapsing MS patients' experiences during treatment with interferon beta-1a. J Neurosci Nurs.

[27] Burton CR (2000). Living with stroke: a phenomenological study. J Adv Nurs.

